# Green synthesis, characterization, antibacterial, and antifungal activity of copper oxide nanoparticles derived from *Morinda citrifolia* leaf extract

**DOI:** 10.1038/s41598-023-46002-5

**Published:** 2023-11-01

**Authors:** Manogar Priya, Raja Venkatesan, Simon Deepa, Siva Sankar Sana, Soundhar Arumugam, Abdulnasser M. Karami, Alexandre A. Vetcher, Seong-Cheol Kim

**Affiliations:** 1 Department of Chemistry, School of Basic Sciences, Vels Institute of Science, Technology and Advanced Studies, Chennai, Tamil Nadu 600117 India; 2 School of Chemical Engineering, Yeungnam University, Gyeongsan, 38541 Republic of Korea; 3 Department of Mechanical Engineering, Indian Institute of Technology Guwahati, Guwahati, Assam 781039 India; 4 Department of Chemistry, College of Science, King Saud University, 11451 Riyadh, Saudi Arabia; 5 Institute of Biochemical Technology and Nanotechnology, Peoples’ Friendship, University of Russia (RUDN), 6 Miklukho‐Maklaya St., Moscow, Russia 117198

**Keywords:** Chemistry, Materials science, Nanoscience and technology

## Abstract

The green methodologies of nanoparticles with plant extracts have received an increase of interest. Copper oxide nanoparticles (CuO NPs) have been utilized in a many of applications in the last few decades. The current study presents the synthesis of CuO NPs with aqueous extract of *Morinda citrifolia* as a stabilizing agent. The leaf extract of *Morinda citrifolia* was mixed with a solution of copper sulphate (CuSO_4_·5H_2_O) and sodium hydroxide as a catalyst. UV–visible spectroscopy, FTIR, XRD, SEM, TEM, and EDAX analysis were performed to study the synthesized CuO NPs. Particle size distribution of the synthesized CuO NPs have been measured with dynamic light scattering. The CuO NPs synthesized were highly stable, sphere-like, and have size of particles from 20 to 50 nm. Furthermore, as-formed CuO NPs shown strong antibacterial activity against the Gram-positive bacteria (*Bacillus subtilis,* and *Staphylococcus aureus*), and Gram-negative bacteria (*Escherichia coli*). CuO NPs revealed a similar trend was analysed for antifungal activity. The zone of inhibition for the fungi evaluated for *Aspergillus flavus* (13.0 ± 1.1), *Aspergillus niger* (14.3 ± 0.7), and *Penicillium frequentans* (16.8 ± 1.4). According to the results of this investigation, green synthesized CuO NPs with *Morinda citrifolia* leaf extract may be used in biomedicine as a replacement agent for biological applications.

## Introduction

Nanotechnology is growing as an essential area with enormous potential for many applications due to the distinctive characteristics of nanoparticles (NPs)^1^. In comparison with their bulk substitutes, these nanoscale materials have enhanced optical, magnetic, catalytic, and electrical capacities^2,3^. As a result, there is more interest in producing sustainable and effective methods for synthesizing nanoparticles. Traditional methods of synthesizing nanoparticles often involve the use of hazardous chemicals, high temperatures, and energy-intensive processes, leading to environmental concerns and potential toxicity. To address these issues, green synthesis has gained considerable attention as a promising alternative. Green synthesis, also known as environmentally friendly or sustainable synthesis, involves the use of natural resources, biomolecules, or environmentally benign materials to fabricate nanoparticles^4–6^. It offers several advantages over conventional methods, including reduced energy consumption, minimal use of toxic chemicals, biodegradability, and the potential for large-scale production^7,8^.

Metal oxide nanoparticles attract the attention of researchers due to the connect bulk and atomic structures. NPs have unique physicochemical characteristics which include significant reactivity, huge surface area, pore size, and particles shape^9^. Introduction to novel nanoparticles might put immunological in nature and inflammation responses to the challenge^10^. The most rapid adopters of nanotechnology are the areas of information and communication (such as electrical and optoelectronic sectors), food technology, energy technology, and medical products (including a number of pharmaceuticals and drug delivery systems, diagnostics, and medical technology). Toxicity arising from nanomaterials might present new problems. These situations may involve nanoparticle which have been introduced into the environment or which were given to individuals via nanotechnology products. Nanoparticles are can be synthesized by physical, chemical, biological, and hybrid procedures^11–13^. Toxic materials render the production of physical and chemical nanoparticles more difficult. Effective eco-friendly biogenetic methods of production have become more common due to their ease of use and flexibility^14,15^. Before, nanoparticles that needed to be produced via chemical and physical methods. Nanoparticles and nanotechnology deal with small materials. Nanoparticles were extensively studied in recent years due to their many potential uses in chemistry, drug delivery, biomedical, and other areas^16–19^.

As a result of their biocidal characteristics, copper nanoparticles are now attractive wounds treatment. With its cheap price and excellent physical and chemical attributes, copper NPs can be utilized in process bandage. The method for the production of nanomaterials is dependent on their small dimensions and high surface-to-volume ratio^20^. Metal and metal oxide nanoparticles have been employed in a wide range of applications. Several distinctive methods for adjusting shape and size includes metal vapour co-deposition, electrochemical reduction, gas phase evaporation, thermal decomposition, radiolytic reduction, and chemical reduction^21–24^. Nanosized particles can be produced with chemical and physical methods like micro-emulsion are immersed. For instance, flame-based aerosol techniques, Sono chemical hydrothermal techniques, solid-state techniques, and the system for producing nanoparticles. Nanoparticles cannot be used in healthcare due to there are generated with toxic materials. Clean, biocompatible, nontoxic, and sustainable nanoparticle processing is thus advantageous^25,26^. This field is currently concentrating on “green” chemistry and bio-processors.

Plants are used in “green synthesis” for the production of metal nanoparticles. Green synthesis in biotechnology and nanotechnology has an opportunity for advantages for the economy and the environment^27^. Green chemistry synthesizes in an environmentally friendly and efficient method. Nanoparticles have been proposed to be synthesized in plants, algae, bacteria, yeast, and fungi^28^. The nanoparticles of copper can be produced from plant extracts using eco-friendly, low-cost, and biocompatible reducing agents^29,30^. Copper oxide nanoparticles development is enhanced with ascorbic acid in *Morinda citrifolia* leaf extract. In addition to their distinctive characteristics, such as a large surface area, catalytic activity, and antibacterial capabilities, CuO NPs have attracted interest in many other fields. Bioengineered CuO NPs are those that undergo CuO nanoparticle synthesis or modification with biological processes like bacteria, fungi, or plant extracts. The significance of using bioengineered CuO NPs lies in their potential to provide more sustainable, efficient, and biocompatible solutions across various fields, from healthcare and environmental protection to materials science and energy.

In this work, we developed an efficient method to synthesize CuO NPs and studied the crystalline nature, chemical composition, and interactions between NPs and the reducing agent. *Morinda citrifolia* leaf extract was used as a stabilizing agent in the green synthesis of CuO NPs. The copper oxide nanoparticles with functional components, structure, and particle size were studied with UV–vis, FTIR, XRD, SEM, TEM, and DLS analysis. Furthermore, the antibacterial effects of the CuO NPs were investigated by Gram-positive bacteria (*Bacillus subtilis,* and *Staphylococcus aureus*), and Gram-negative bacteria (*Escherichia coli*) with the agar diffusion method.

## Materials and methods

### Materials

Copper sulphate (CuSO_4_·5H_2_O) was purchased from Sigma-Aldrich (98%). Hydrochloric acid (HCl) (35%), sodium hydroxide (98%) was used to monitoring the pH, were received from Merck. The leaves of *Morinda citrifolia* have been collected in Chennai, Tamil Nadu. The dissolution of 2.5 g of CuSO_4_·5H_2_O in 100 mL deionized water yielded a 1 × 10^–2^ M stock solution of copper sulphate. Bacterial and fungal cultures were grown in the medium, including *Bacillus subtilis*, *Staphylococcus aureus*, *Escherichia coli*, *Aspergillus flavus*, *Aspergillus niger*, and *Penicillium frequentans*. All of the chemical and solvents utilized were of analytical grade.

### Preparation of *Morinda citrifolia* leaf extract

**Figure 1:**
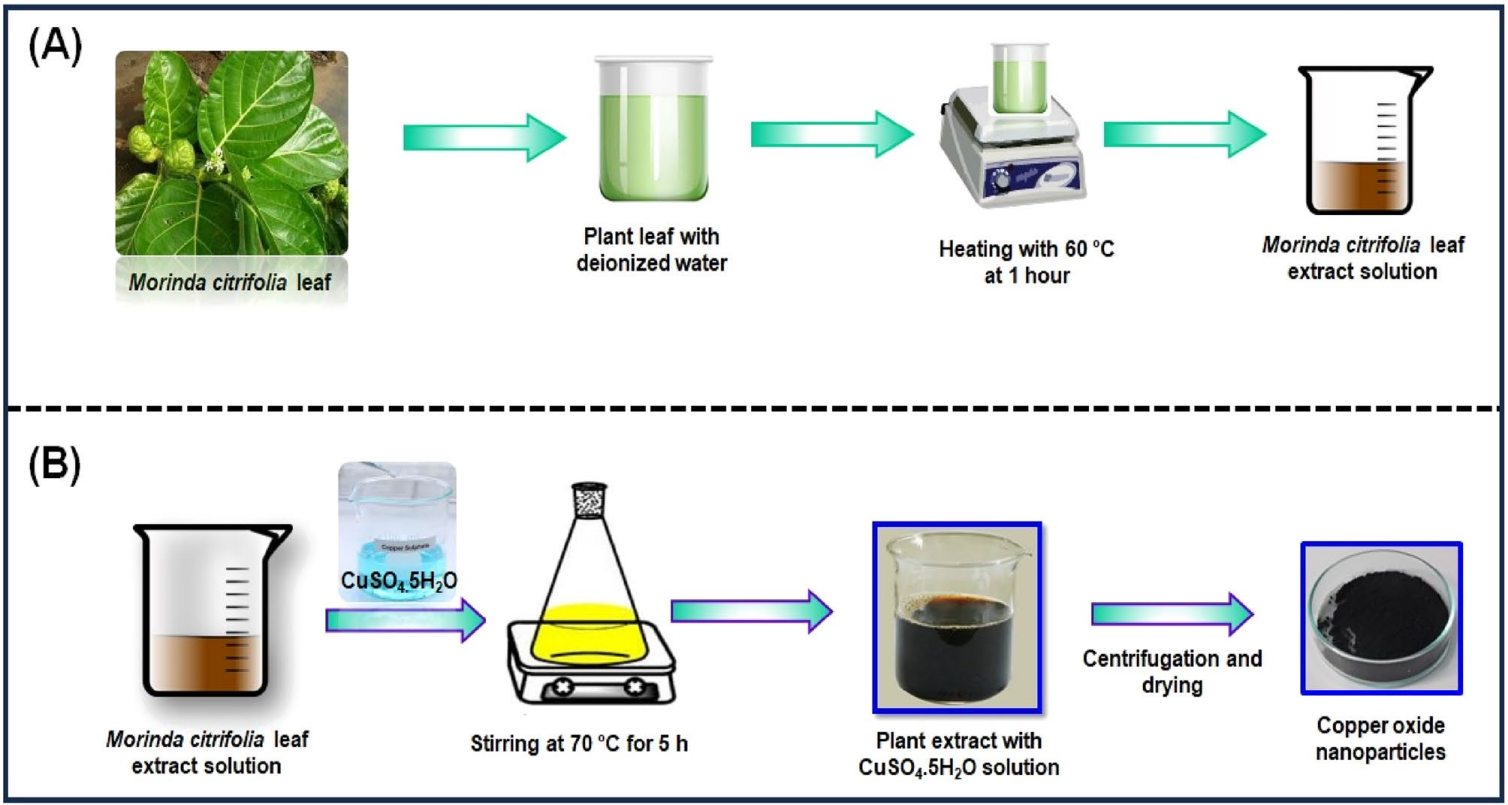
(**A**) Schematic representation of eco-friendly synthesis of copper oxide nanoparticles using *Morinda citrifolia* leaf extract; (**B**) Schematic diagram of CuO NPs from leaf extract of* Morinda citrifolia.*

The *Morinda citrifolia* leaf extract can be seen in Fig. 1A. *Morinda citrifolia*, a plant of the *Rubiaceae* family, had its leaves collected from a garden in Chennai. We weighed and cleaned *Morinda citrifolia* leaves several times with tap water and deionized water after collecting to get rid of any extra dust or contaminants. After that, a slice the leaf in small pieces, add 100 mL of distilled water, and immerse the mixture in a water bath heated to 60 °C for 1 h. The green extract can be processed in a burette and used as a reducing or capping agent. The extract was kept at 4 °C for further studies.

### Synthesis of CuO NPs from *Morinda citrifolia* leaf extract

Figure 1B shows the synthesis of CuO NPs from *Morinda citrifolia* leaf extract solution. 2.5 g of CuSO_4_·5H_2_O was dissolved in 100 mL of Deionized water (DI) to initiate the green synthesis process for CuO NPs. After, 50 mL of *Morinda citrifolia* extract solution to 100 mL of 1 × 10^–2^ M CuSO_4_·5H_2_O solution, the pH was kept at 7.0 with NaOH. The solution then underwent to a reflux at a magnetic stirrer. The colour of the solution changed as it was stirring with a from pale-green to a deep-brown while maintaining for 5 h at 70 °C. After centrifuging the solution for 24 h, it was filtered. The solid precipitate was washed three times with deionised water, followed by an 100% ethanol wash for CuO NPs separation, dried at 60 °C for 4 h, and kept at 4 °C for further application.

The following equations explain the synthesis mechanism for CuO NPs;

1 $$ {\text{Cu}}^{{{2} + }} \left[ {{\text{CuSO}}_{{4}} \cdot {\text{5H}}_{{2}} {\text{O}}} \right] + {\text{Extract }}\left( {{\text{From}}\,Morinda\,citrifolia\,{\text{extract}}} \right) \to \left[ {{\text{Cu}} - {\text{Extract}}} \right]^{{{2} + }} $$

2 $$ \left[ {{\text{Cu}} - {\text{Extract}}} \right]^{{{2} + }} + {\text{NaOH}} \to \left[ {{\text{Cu}}\left( {{\text{OH}}} \right)_{{2}} - {\text{Extract}}} \right] $$

3 $$ \left[ {{\text{Cu}}\left( {{\text{OH}}} \right)_{{2}} - {\text{Extract}}} \right] + {\text{Stirring}} \to \left[ {{\text{CuO}}} \right]{-}{\text{Precipitate}} \to {\text{Heating}} \to {\text{CuO}}\,{\text{NPs}} $$

### Characterization of synthesized CuO NPs

The UV–Visible spectrum of effectively obtained CuO NPs was collected with an (*Oceian optics JAZ, USA*) spectrophotometer. The UV spectrum of copper oxide nanoparticle synthesis in colloidal solution was observed at wavelengths ranging from 200 to 800 nm. The FTIR spectrometer (*Perkin Elmer, Spectrum-2, USA*) with KBr pellet was used for collecting functional group data in the region of 4000–400 cm^−1^. The FTIR spectrum of obtained CuO NPs was examined. Different modes of vibration in the CuO NPs have been identified and assigned to evaluate the presence of different functional groups that aid the extract of the *Morinda citrifolia* plant. XRD measurement of the CuO NPs, where only 5.0 ml of the extract was added, was done on a Shimadzu XRD-6000 diffractometer operating at a voltage of 40 kV and current of 20 mA with Cu-Kα radiation (λ = 1.54 Å). The XRD spectrum has been examined and acquired with scanning range values of 20° and 80°.SEM study of the surface morphology of CuO NPs was performed (*CARL ZEISS, Jena, Germany*). The inner morphology of the CuO nanoparticles was studied with *Morinda citrifolia* extract, and images were captured using a TEM (*JEOL, JEM-2100, Japan*). For descriptive purposes, a 5.0 ml of the materials were sonicated in ethanol, and a drop of it was cast in a copper grid with a 300-mesh carbon layer by layer for magnetic measurements. The particle size distribution (PSD) of the synthesized CuO NPs have been measured with the Dynamic Light Scattering (DLS) measurements instrument's standard operating procedure.

#### Antibacterial and antifungal studies

##### Methodology

*Bacillus subtilis* (MTCC6133), *Escherichia coli* (MTCC6133), and *Staphylococcus aureus* (MTCC96) were collected from the Microbial Type Culture Collection and Gene Bank (MTCC), Institute of Microbial Technology, Chandigarh, India. Standard cultures of bacteria have been sub-cultured into newly prepared nutrient agar and incubated at 37 °C for 24 h for the production of fresh cultures of bacteria. Marina Labs Research and Development offers fungal cultures of *Aspergillus flavus* (MLAC1101), *Aspergillus niger* (MLAC1201), and *Penicillium frequentans* (MLAC 2101). The fungi were sub-cultured for 72 h to produce the sporulation process and the developing spore were examined for antifungal activity.

##### Assay for antibacterial activity by well diffusion

The zone of inhabitation method was employed to evaluate the antibacterial activity of the offered materials^31–33^. Mueller–Hinton agar plates were applied to test the samples. The agar plate was streaked with the different cultures (bacterial strains). Then, using a sterile cork-borer, 5 mm diameter wells were cut into the agar medium. For 20 min, the plates are allowed to dry to remove all remaining moisture. The compounds of 15 µL, 20 µL, and 25 µL were administered into each well. As a positive control, a well containing 15 µL of streptomycin antibiotic was used. The plates were incubated at 37 °C. The tests were performed in duplicates. Every plate was evaluated for zones of inhibition 24 h after incubation. The diameter of the inhibitory zone was calculated in millimetres (mm).

##### Assay for antifungal activity by well diffusion

For testing the antibacterial activity of the offered sample, the agar well diffusion method was employed. Sabouraud’s Dextrose agar plates were employed for testing the specimens. The agar plate’s surface was streaked with the different cultures (fungal strains). The agar medium was then cut into 5 mm diameter wells with a sterile cork-borer. For 20 min, the plates are allowed to dry to remove additional moisture. Compounds of 15 µL, 20 µL, and 25 µL were dispensed into each well, with 5.0 mg of Fluconazole serve as a positive control. At 37 °C, the plates were incubated. The tests have been carried out in duplicate. After 24 h of incubation, each plate was examined for zones of inhibition. The zone of inhibition was recorded as the diameter of inhibition zone in mm.

#### Leaves collection permission

The *Morinda citrifolia* leaves have been obtained from Chennai, Tamil Nadu in India, and all of the national guidelines, legislation and/or protocols have adhered appropriately. *Morinda citrifolia* is a flora species found predominantly in India. In Tamil Nadu, this species is a very common tree seen in road sides and in every gardens. Hence, the usage of this plant needs no permission and licensing.

### Ethical approval

We comply with relevant guidelines and legislation regarding the sample collection in the present study. The plant leaves (*Morinda citrifolia*), in the present study is not endangered. In 2023, leaves of the *Morinda citrifolia* plant were collected in Chennai, Tamil Nadu, India. There are no plant material samples for the current study.

### Consent to participate

All person named as author in this manuscript have participated in the planning, design and performance of the research and in the interpretation of the result.

## Result and discussion

The change in the colour of the reaction solution suggests the synthesis of CuO NPs by the reduction of CuSO_4_·5H_2_O during treatment with extracts of *Morinda citrifolia* leaf. The change in color of the reaction solution after 2 h reveals the synthesis of CuO NPs. The result indicates that the Cu-Extract^2+^ ions in the reaction mixture have changed to copper oxide with nanometric size. In the synthesis of CuO NPs, different types of plant extracts are used as reducing and stabilizing agents. The resultant nanoparticles have no surface instead of encased in a medium or gel, and their catalytic and other characteristics can be restricted, while particle stabilized and microgel stabilized nanoparticles characteristics may be altered by modifying the temperature and pH. Table 1 presents the green synthesis of CuO NPs with different plants.

**Table 1:** Summarises the bio synthesis of copper oxide nanoparticles using several plants and the acquired particle size.

S. No.	Precursors	Reducing agents	Particle size (nm)	Refs.
1	Cu(CH_3_COO)_2_	*Kigelia africana*	70.0–100.0	^34^
2	CuSO_4_	*T. arjuna*	23.0	^35^
3	CuSO_4_·5H_2_O	*Pleurotus citrinopileatus*	60.0–70.0	^36^
4	CuSO_4_·5H_2_O	*Rhuscoriara L*	19.0	^37^
5	CuSO_4_	*Seedless dates*	78.0	^38^
6	Cu(NO_3_)_2_	*Gloriosa superba L*	5.0–10.0	^39^
7	CuSO_4_	*A. indica*	26.0–30.0	^40^
8	CuSO_4_	*Ginger roots*	60.0	^41^
9	CuSO_4_	*Morinda citrifolia*	28.2	^42^
10	CuSO_4_·7H_2_O	*Bunium persicum*	50.0	^43^
11	CuSO_4_·5H_2_O	*Syzygium cumin*	10.0	^44^
12	CuSO_4_	*Cissus vitiginea*	10.0–20.0	^45^
13	CuSO_4_·5H_2_O	*Native cyclodextrin*	20.0	^46^
14	CuSO_4_·5H_2_O	*Tilia extract*	4.7–17.4	^47^
15	CuSO_4_·5H_2_O	*Celastrus paniculatus*	2.0–10.0	^48^
17	CuSO_4_·5H_2_O	*Grewia asiatica L*	2.0	^49^
18	CuSO_4_	*Vaccinium corymbosm*	3.0–12.0	^50^
19	CuSO_4_	*Carum carvi*	12.4	^51^
20	CuSO_4_	*Spinacia oleracea*	134.8	^52^
21	Cu(NO_3_)_2_·3H_2_O	*Hibiscus cannabinus*	450.0–900.0	^53^
22	CuSO_4_·5H_2_O	*M. piperita*	100.0	^54^
23	CuCl_2_·2H_2_O	*Vaccinium*	400.0	^55^
24	CuSO_4_·5H_2_O	*Morinda citrifolia*	29.2	Present work

### UV–Visible spectroscopy

The CuO NPs were investigated with UV–Visible spectroscopy to identify the optical band gap. A distinctive peak was found at 256 nm, which might be assigned to surface plasmon resonance (SPR), and it was revealed. The SPR at 256 nm indicates the synthesis of CuO NPs. SPR occurred as a result of an oscillation of surface electron of nanoparticles, so this result agreed with earlier research^34^. In accordance with Mie's theory, the quantity of SPR bands is mainly determined by the shape of nanoparticles that are produced. The spherical form of the nanoparticle is mostly because of a single SPR band. With equation, the band gap energy similar to the wavelength of peak absorption was calculated. The band gap energy can be calculated with the formulas below.

4 $$ {\text{Eg}} = {\text{hvg}} = {\text{hc}}/\lambda {\text{g}} $$

where h is the plank constant, C is the velocity of light, Eg is the energy gap, and g is the measured absorption wavelength.

**Figure 2:**
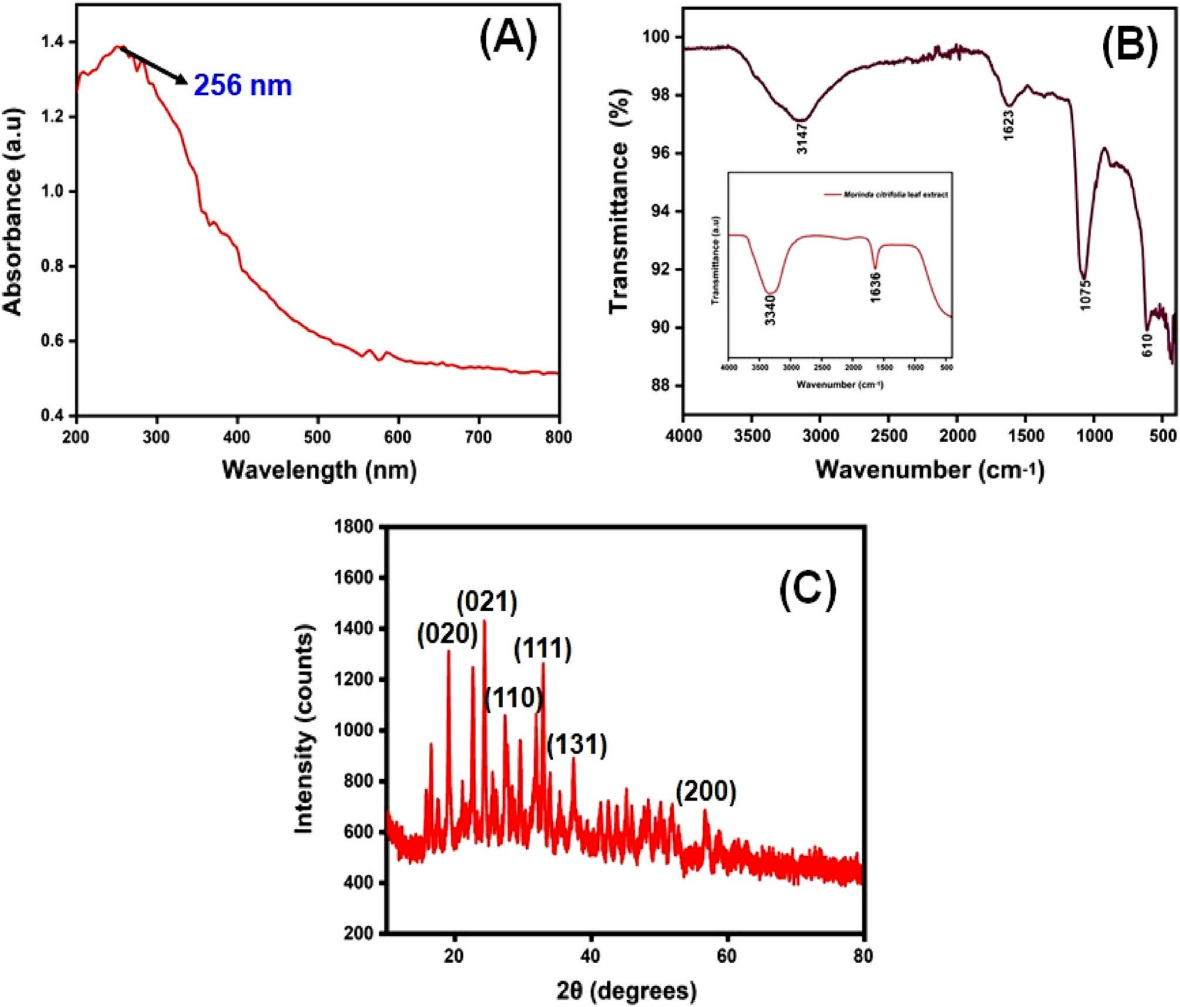
(**A**) UV–visible spectrophotometer results of synthesized CuO NPs; (**B**) FTIR spectra and (**C**) XRD pattern.

The synthesized Cu nanoparticle’s strongest and most sharp absorption peak appears at 256 nm, and it shows the blue shift absorption observed in Fig. 2A. The calculated band gap energy from the UV–visible absorption spectrum is 1.006 eV^56,57^. The decrease in particle size has been triggered with a shift in absorption towards smaller wavelengths.

### FTIR spectral analysis

The FTIR spectrum of ecofriendly obtained plants extracts and CuO NPs were studied. The spectra were collected between 4000 and 400 cm^−1^. A type of vibrations in the CuO materials have been determined and assigned to identify the existence of different functional groups that aid with the chemical reduction. The FTIR spectra of the plant extract *Morinda citrifolia* are shown in (Fig. 2B inset), consistent with the earlier research^42^. The absorption bands at 3340 cm^−1^ correspond to the absorption band of –OH functional group. The significant peaks at 1636 cm^−1^ suggest the existence of a functional group denoted as –NO_2_ in the plant extract The *Morinda citrifolia* leaf plant served in the reduction of copper ions as well as the capping of CuO. Figure 2B presents the results of the research study performed on the peaks. For O–H stretching of water and –C=O stretching of aldehydes and ketones, the major peaks are observed at 3147 cm^−1^ and 1623 cm^−1^, respectively^58,59^. The stretching vibration peak for the C=H and H–C–H functional groups is at 495 cm^−1^, and the stretching vibration peak for the C=H and H–C–H functional groups is at 2923 cm^−1^, confirmation the presence of synthesized CuO nanoparticles in the materials^60^.

### XRD analysis

XRD measurements revealed the crystalline characteristics of the obtained copper nanoparticles. The XRD spectrum of the synthesized copper nanoparticles is presented in Fig. 2C. The CuO NPs exhibited crystalline XRD peaks at 2θ values of 19.03°, 24.36°, 27.39°, 32.99°, 37.55°, and 56.74° which correspond to the planes of crystals of (020), (021), (110), (111), (131) and (200), respectively. The plane alignments of the synthesized CuO NPs were in excellent accordance with the standard CuO nanoparticles obtained for the International Centre of Diffraction Data Card (JCPDS No.: 00-041-0254). The XRD pattern suggested that the synthesized CuO nanoparticles are polycrystalline in characteristic and resembled the monoclinic tenorite phase of the CuO structure. Lattice parameters are α = 4.79 Å, the intensities and positions of peaks are in moral promise with the stated standards^61,62^. Additionally, the well- distinct and sharp CuO images detected from XRD patterns approves the moral crystalline nature of the green synthesized CuO NPs. Comparable results were also stated in earlier like works^63,64^. Strong orientation and broad diffraction bands in the XRD spectrum can be attributed to the nano dimensional conditions of the obtained nanoparticles. In addition, the XRD pattern indicated the newly synthesised nanoparticles are nanocrystalline. The average crystallite size of CuO nanoparticles was calculated using a Debye–Scherrer formula (Eq. 5).

5 $$ {\text{D}} = \frac{{{\text{K}}\lambda }}{\beta cos\theta } $$

where D is the average diameter of the nanoparticles, K is the Sherrer constant, λ is the wavelength of x-ray diffraction (015,406 nm), β is the full width at half maximum, and θ is Bragg angle (degree).

The average crystalline size of synthesized CuO nanoparticles has been estimated to be in the range of 25–30 nm using the Debye Scherrer formula, and the crystal structure of synthesised CuO nanoparticles has been shown to be face-cantered cubic structure.

### SEM analysis

**Figure 3:**
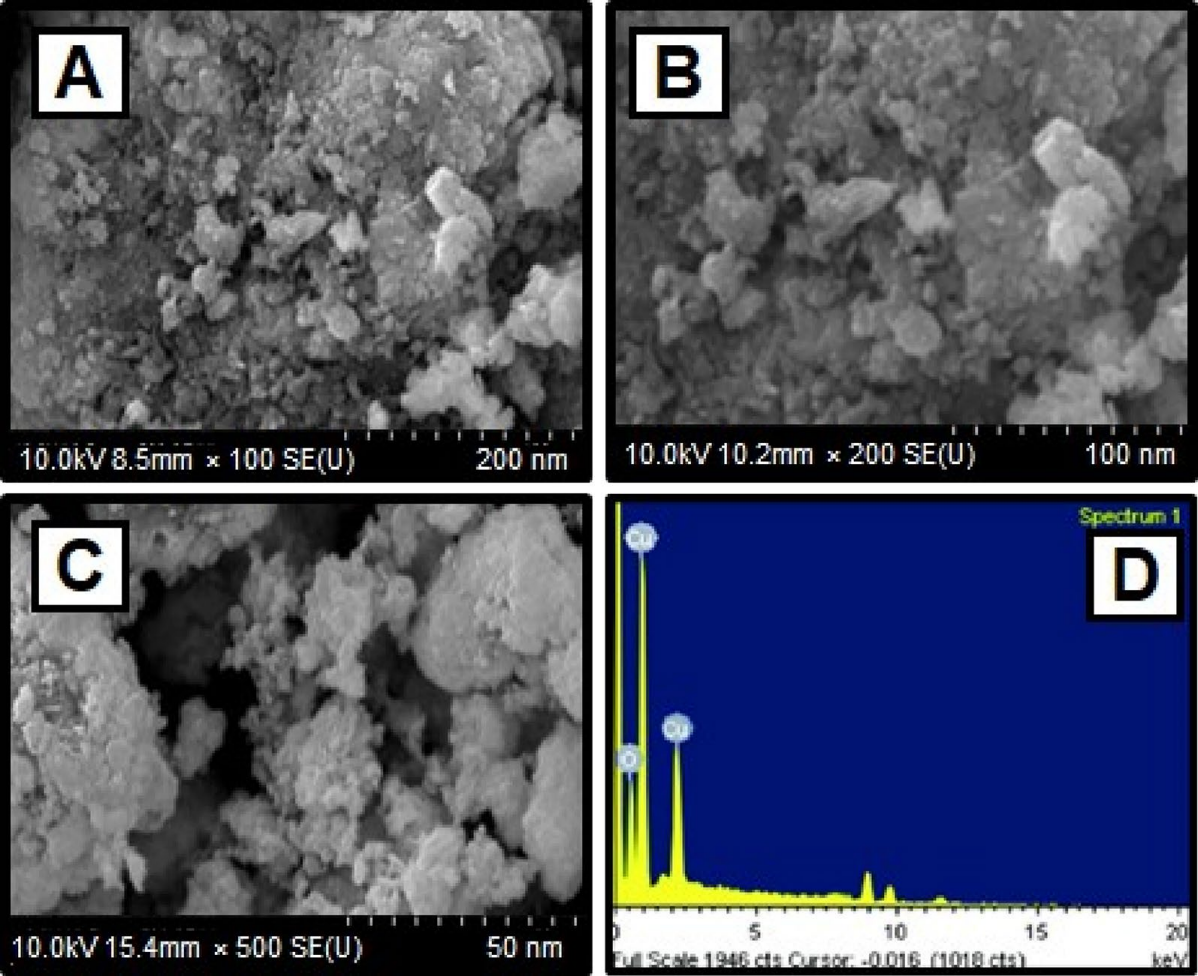
(**A**)–(**C**) SEM image of CuO NPs synthesized using CuSO_4_·5H_2_O and *Morinda citrifolia* leaf extract (**D**) EDX analysis of CuO NPs.

The scanning electron microscope (SEM) confirmed the size and structure of the nanoparticles that were synthesized. The images from SEM suggest that the green synthesized CuO NPs have a major distribution and spherical shapes^65^, and have an average size of 29.2 nm. As predicted, agglomerations decreased as the size of particles increased, due to size of particles increased gain size linear. When the agglomeration of particles can be attributed to an effort to decrease surface free energy, SEM images of CuO nanoparticles are showed in Fig. 3A–C. The surface alternatives are clearly shown, paying special attention to the fact that nanoparticles were synthesized.

The elemental composition of CuO NPs produced with green synthesis method have been identified using a EDAX device. The elemental composition of CuO nanoparticles can be seen in Fig. 3D. The elements are copper (65%), oxygen (23%), and carbon (12%) shown the Table 2. The high concentration of copper metal in the advanced levels indicates the synthesis of CuO NPs via a green methodology.

**Table 2:** Elemental composition (EDAX analysis) of the green synthesized CuO nanoparticles.

S. No.	Element	Weight (%)	Atomic (%)
1	O	22.8	53.9
2	Cu	64.9	43.7
3	C	11.9	2.0

### TEM analysis

**Figure 4:**
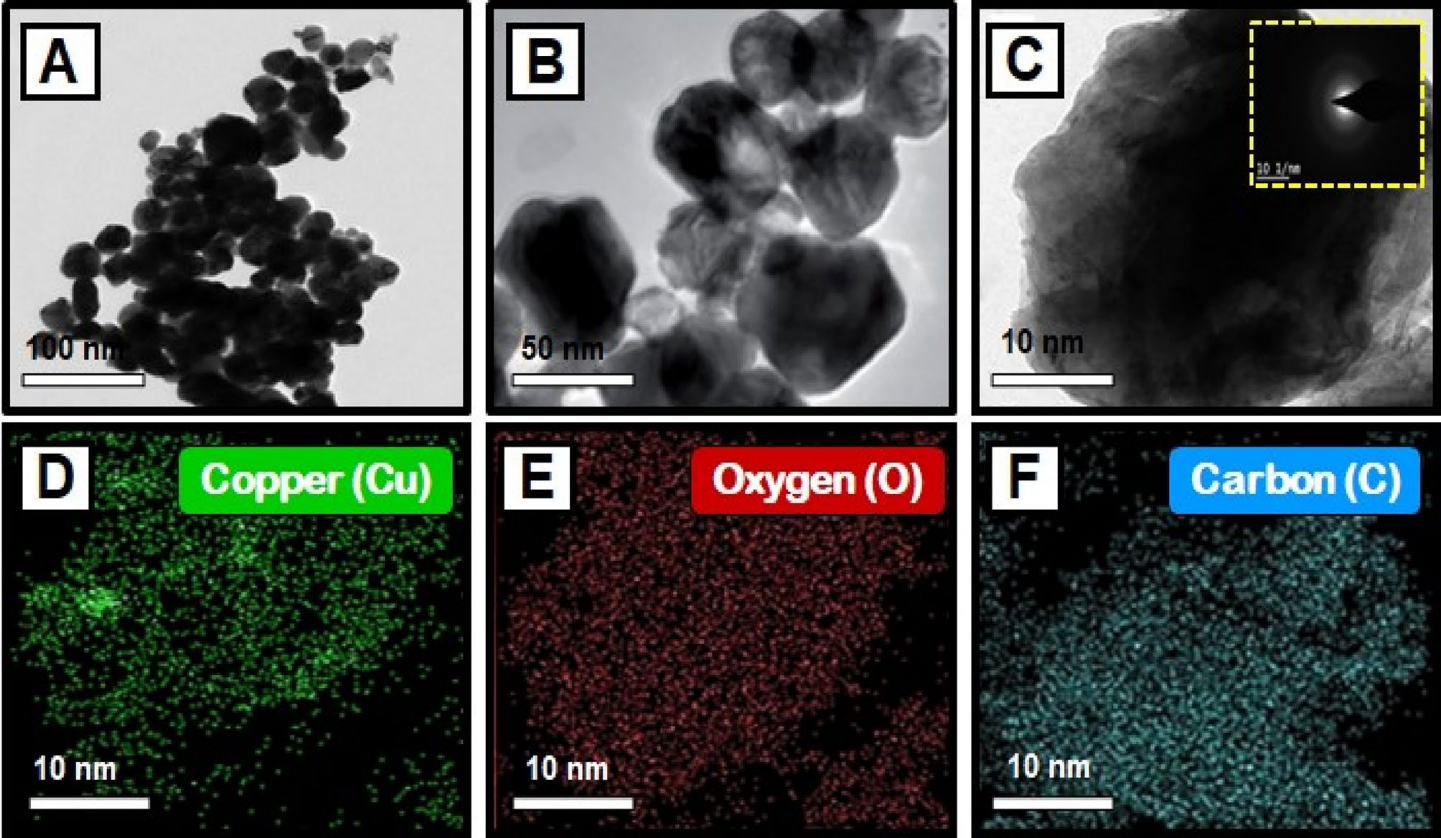
(**A**–**C**) TEM images of copper oxide nanoparticles, and SAED image of CuO NPs [insets Fig. (**C**)]; Elemental mapping analysis of CuO NPs from *Morinda citrifolia* leaf extract, (**D**) Copper, (**E**) Oxygen, and (**F**) Carbon elements.

The TEM images of synthesized CuO nanoparticles are shown in Fig. 4A–C. TEM was employed to study the particle size and surface morphology of *Morinda citrifolia*-mediated CuO NPs, and the results suggested that the CuO were polydisperse and cylindrical in structure. The SAED pattern confirmed the crystal structure of CuO NPs. SAED patterns suggest that CuO NPs have distinctive lattice fringes which are similar with the normal CuO structures and have excellent crystalline quality. Padmavathi et. al., observed that produced CuO NPs are surface elements and can serve as a successful reducing agent of CuO ions to CuO NPs in *Morinda citrifolia* extract^66^. Sodium hydroxide as a catalyst agent, inhibiting CuO NPs aggregation. The TEM results of CuO NPs were fully consistent with the XRD pattern of obtained CuO NPs. This study was aided by the results of Fardood et al., which noticed the FCC structure of CuO NPs using the TEM and SAED patterns of CuO NPs synthesized from *Morinda citrifolia* leaf extract^67^. The corresponding SAED pattern (inset in Fig. 4C) indicates that the copper particles given among the CuO NPs are highly crystalline and have the predicted alignment. The Cu, O, and C elements are seen in the element mapping images of the synthesized CuO NPs (Fig. 4D–F). The presence of nanoparticles in the material is evident as Cu, O, and C are confirmed with synthesized CuO NPs.

### The particle size distribution of CuO NPs

**Figure 5:**
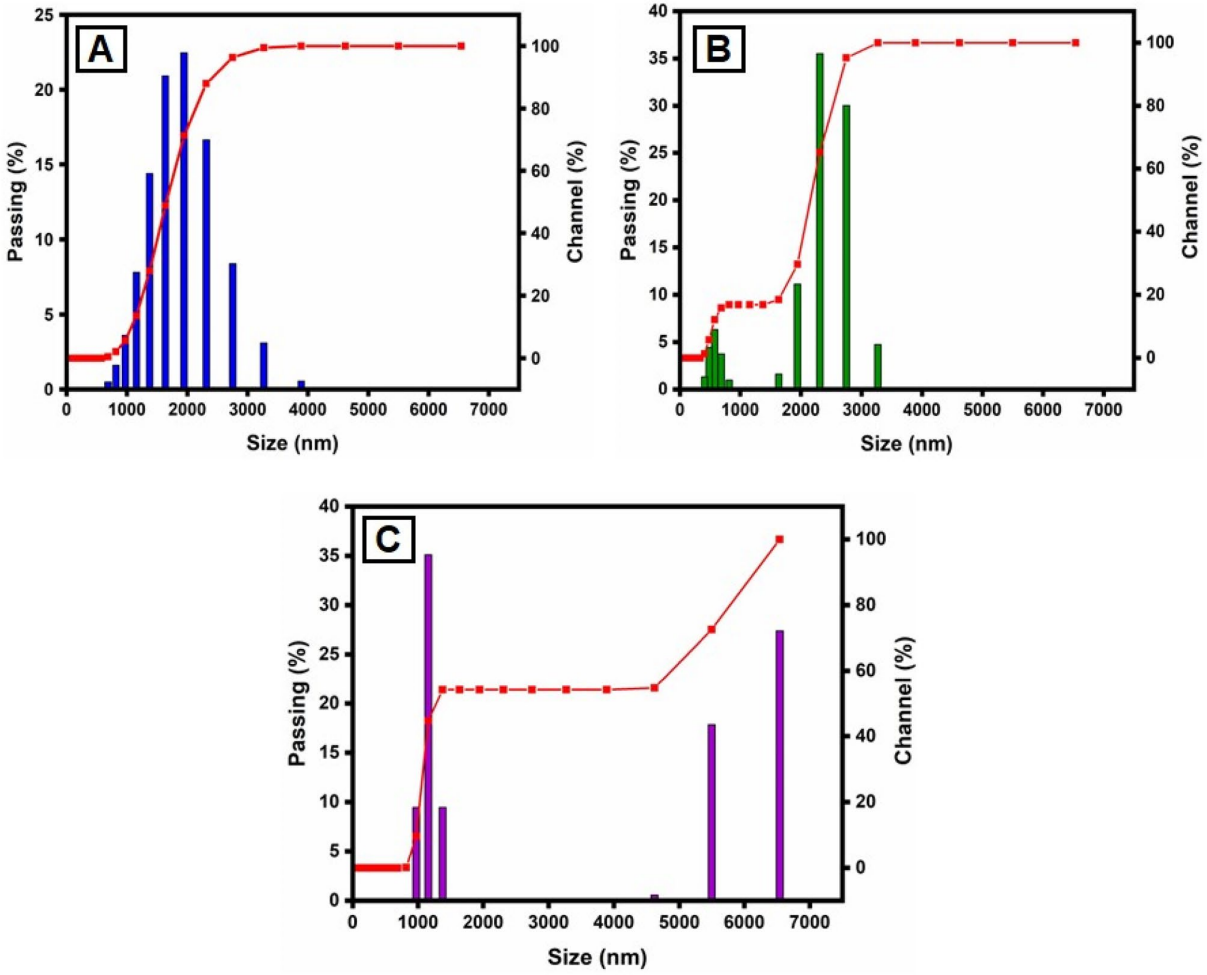
Particle size distribution (PSD) of the synthesized CuO NPs by the DLS methods, for varying CuSO_4_·5H_2_O concentration: (**A**) 15 µL; (**B**) 20 µL and (**C**) 25 µL.

This method is utilized for synthesizing particles with colloidal structure. The particle size distribution (PSD) for colloids produced at different concentrations of CuSO_4_·5H_2_O and constant *Morinda citrifolia* content, as measured with the dynamic light scattering (DLS) method, is shown in Fig. 5. For the three samples, the types of distribution and average diameters changed. The sample prepared with 1 × 10^–2^ M CuSO_4_·5H_2_O is the most monodisperse and has an average diameter of about 100.0 nm, however the samples obtained with 15, 20 and 25 µL, despite having average dimensions of 49.1 nm, 37.0 nm and 29.2 nm, respectively, have more polydispersity suggested that 15 µL given the best performance. The results reported in previous articles^68,69^, comparing Fig. 5A–C indicates the concentration of the copper sulphate greatly impacts the size distribution of the nanoparticles. Aside from the formation of smaller particles, it was expected that a lower CuSO_4_.5H_2_O concentration would result in a narrower size distribution since the ratio of *Morinda citrifolia*:Cu^2+^ ions would be greater in this case. However, the DLS data shown a trend toward the reverse site. As the concentration of CuSO_4_·5H_2_O decreased, the size distribution expanded. The result can be addressed if we understand that the average diameter measured with DLS results from nanoparticles surrounded by *Morinda citrifolia* rather than “naked” CuO NPs. In addition *Morinda citrifolia* molecules may attach to the surface of particles at lower concentrations of copper sulphate due to the higher *Morinda citrifolia*:Cu^2+^ ions ratio^70,71^. *Morinda citrifolia* molecules may form more than one layer on the nanoparticles. The outermost layer can absorb water, producing tumescence of the composite nanoparticles and, as a result, increasing particle sizes.

### Antibacterial activity

**Figure 6:**
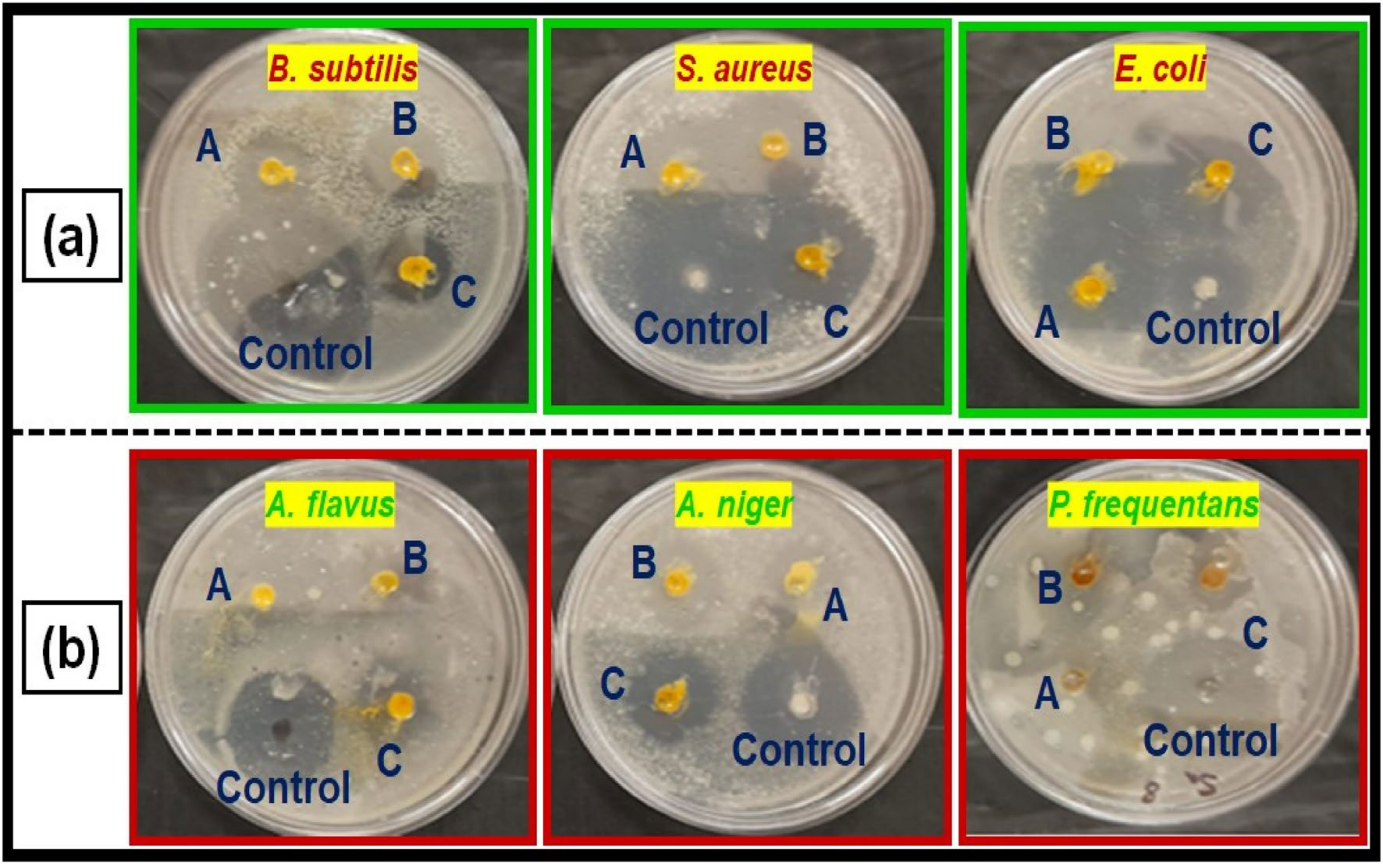
(**a**) Antibacterial activity, and (**b**) antifungal activity of copper oxide nanoparticles from *Morinda citrifolia* leaf extracts; (A) 15 µL, (B) 20 µL and (C) 25 µL; and control of CuO NPs.

The disk diffusion method was used to study the antibacterial activity of CuO NPs against gram-positive and gram-negative pathogenic bacteria such as *B. subtilis, S. aureus*, and *E. coli* (Fig. 6a). In laboratories, nutritional broth has been commonly utilized for sustaining live pathogens of bacteria (as subcultures with 0.5 Mc turbidity) cultivated overnight at 37 °C^72^.

**Figure 7:**
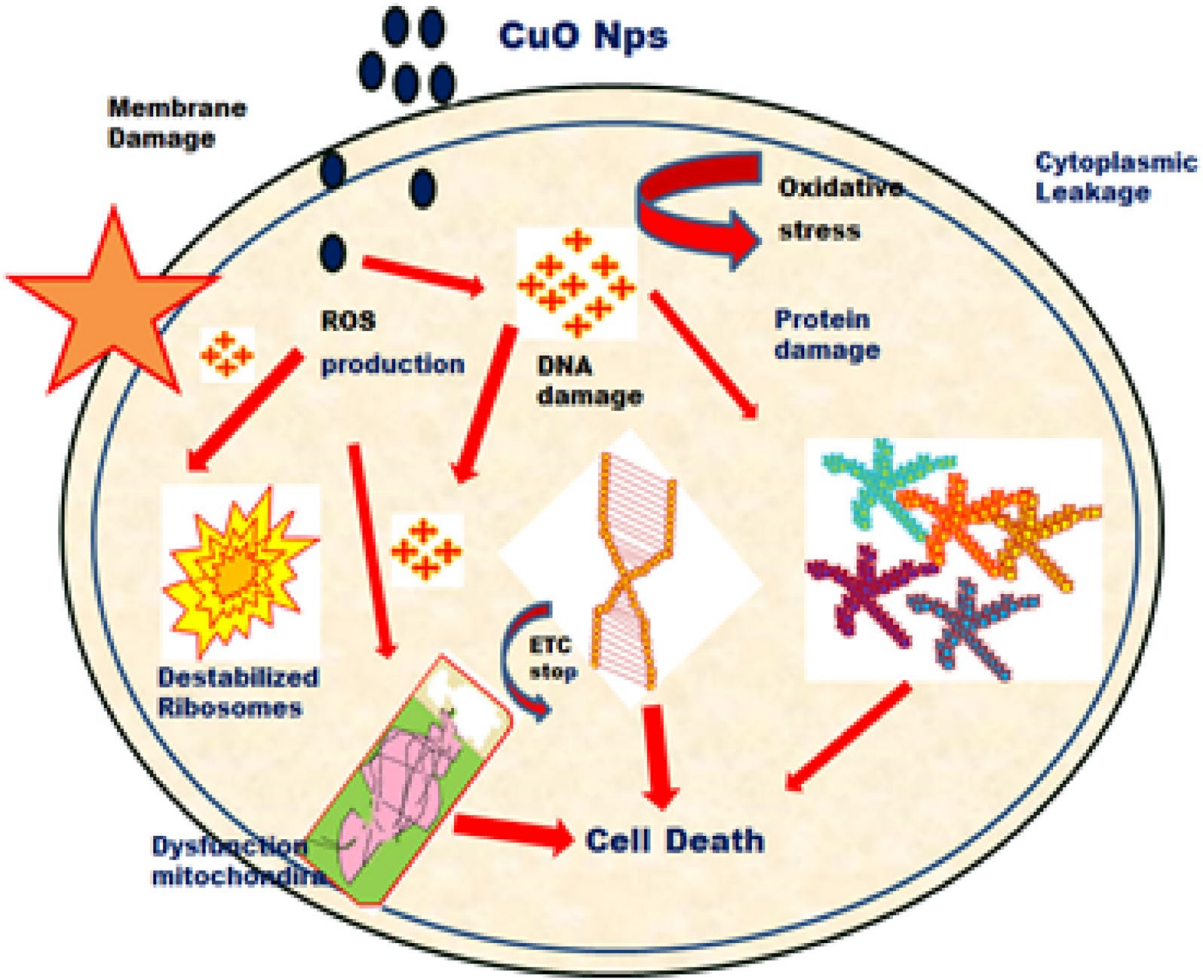
Schematic representation of green synthesis of copper oxide nanoparticles using *Morinda citrifolia* leaf extract.

The fresh bacterial culture was swiped evenly on sterilized Petri dishes with nutrient agar. On the clean disks, synthesized CuO NPs (15, 20 and 25 µL) and an aqueous (*Morinda citrifolia*) leaf extract (25 µL) was poured. As a positive control, 25 mL of chloramphenicol disks were maintained, and all plates were incubated overnight at 37 °C for 24–48 h to identify the development of bacterial inhibition zone surrounding the surface of the disk. The results revealed that the CuO NPs has showed antibacterial activity against the bacteria, *Bacillus subtilis.* It has recorded 13.0 mm zone of inhibition at the concentration of 25 µl*.* However, there was no zone recorded for the bacteria, *Escherichia coli.* The compound showed less activity against the bacteria, *Staphylococcus aureus.* The zone of inhibition recorded for the bacteria, *Bacillus subtilis* (13.6 ± 1.1), *Staphylococcus aureus* (13.2 ± 0.2), and *Escherichia coli* (13.1 ± 1.2) respectively. The antibacterial activity mechanism of green synthesized CuO NPs is shown in Fig. 7. The antibacterial activity mechanism of copper oxide nanoparticles is dependent on the size, structure, and concentration of copper oxide. The three major ways that antibacterial activity follows are as follows. (1) Degeneration of the cell wall and membrane, (2) Infiltration and cellular disruption, and (3) Oxidation stress^73–75^. The antibacterial activity recorded against each individual bacteria for the CuO nanoparticles is presented in Table 3.

**Table 3:** Antibacterial and antifungal activity of CuO NPs against isolated pathogenic bacteria.

S. No.	Microorganisms	Antimicrobial activity—zone of growth inhibition (mm)			
		Control (*Streptomycin*)	CuO NPs (15 µL)	CuO NPs (20 µL)	CuO NPs (25 µL)
1	*Bacillus subtilis*	28.0	7.5	11.2	13.6
2	*Staphylococcus aureus*	26.3	7.6	10.8	13.2
3	*Escherichia coli*	27.8	7.0	11.4	13.1

Microorganisms		Antifungal activity—Zone of growth inhibition (mm)			
		Control (*Flucanazole*)	CuO NPs (15 µL)	CuO NPs (20 µL)	CuO NPs (25 µL)
4	*Aspergillus flavus*	21.4	–	7.6	13.1
5	*Aspergillus niger*	13.7	–	9.2	14.7
6	*Penicillium frequentans*	22.0	7.2	12.3	16.2

### Antifungal activity

Figure 6b shows a similar pattern for CuO nanoparticle’s antifungal activity. The zone of inhibition recorded for the fungi, *Aspergillus flavus* (13.1 ± 1.1), *Aspergillus niger* (14.7 ± 0.7), and *Penicillium frequentans* (16.2 ± 1.4) respectively. However antifungal activity for the fungus, *A*. *niger* was similar to that of control (*Flucanazole*)^76,77^. The antifungal activity recorded for the CuO NPs against each individual fungal species is presented in Table 3.

## Conclusions

The copper oxide nanoparticles were synthesized with an eco-friendly methodology obtained from plant extracts such as *Morinda citrifolia*. The size, shape, elemental composition, and structure of the synthesized CuO NPs were characterized by UV–visible spectroscopy, FTIR, XRD, SEM, TEM and DLS. Within the process of synthesis, the UV–visible absorption spectrum reveals a blue shift as the percentage of plant extract in the resultant mixture rises. XRD patterns suggest that the crystallites of the CuO NPs that developed have a centre cubic structure. The SEM image of the synthesized CuO NPs suggests that these particles exhibit a spherical structure with an average size of the NPs was 29.2 nm. In addition, structural and size studies reveal that CuO NPs synthesized by *Morinda citrifolia* have a high surface-to-volume ratio. In the EDAX spectrum, the elemental percentage of copper in the CuO NPs was found to be highly uniform. However, the results of bacterial activity showed that the CuO NPs acted well. The synthesized CuO NPs has antibacterial activity against *Bacillus subtilis*, *Escherichia coli*, and *Staphylococcus aureus*. According to the results, CuO NPs is more effective than the other two against *Bacillus subtilis*. CuO NPs has been proven to be efficient against three distinct types of fungi: *Aspergillus flavus*, *Aspergillus niger*, and *Penicillium frequentans*. Copper oxide nanoparticles has been shown to be most effective against all three kinds of fungi based on the data: *Aspergillus flavus*, *Aspergillus niger*, and *Penicillium frequentans*. As a result, the data show that the antifungal activity of the green synthesised CuO NPs has a higher than its antibacterial activity. This study suggests that the synthesized CuO NPs could be employed in the biomedical, fuel cell, battery and food storage industries. However, more study should be done on minimize the toxicity of CuO NPs though maintaining and improving their biological efficiency in order to promote the biomedical uses of CuO NPs.

## Data Availability

All data generated or analysed during this study are included in this published article.
